# Long-Term Preservation of Testicular Tissue Integrity and Viability Using Vitrification in the Endangered Black-Footed Ferret (*Mustela nigripes*)

**DOI:** 10.3390/ani10101865

**Published:** 2020-10-13

**Authors:** David Baruc Cruvinel Lima, Lúcia Daniel Machado da Silva, Paul Marinari, Pierre Comizzoli

**Affiliations:** 1 Laboratory of Carnivore Reproduction, School of Veterinary Medicine, State University of Ceará (Universidade Estadual do Ceará, UECE), 1700 Doutor Silas Munguba Avenue, Fortaleza 0714-903 CE, Brazil; davidbarucvet@gmail.com (D.B.C.L.); lucia.daniel.machado@hotmail.com (L.D.M.d.S.); 2 Smithsonian Conservation Biology Institute, National Zoological Park, Front Royal, VA 22630, USA; Marinarip@si.edu; 3 Smithsonian Conservation Biology Institute, National Zoological Park, Washington, DC 20008, USA

**Keywords:** cryopreservation, black-footed ferret, endangered species, testicular tissue, spermatogenesis

## Abstract

**Simple Summary:**

In addition to semen preservation, systematic cryo-banking of testicular tissues is critical to preserve the genetic value of recently deceased or neutered black-footed ferrets. Mature sperm cells produced from vitrified-warmed tissues could be used for in vitro fertilization over multiple generations, which would enhance the genetic management of this rare and endangered species. The objective of the study was to evaluate structural and functional properties of vitrified testicular tissues directly after warming or after warming plus a short period of in vitro culture. Fresh, vitrified/warmed, and warmed/cultured tissues from five adults were analyzed through histology, DNA fragmentation, cell survival, and germ cell composition. Percentages of intact seminiferous tubules decreased right after vitrification/warming and improved after culture (reaching same percentages as the fresh controls). While proportions of cells with intact DNA and viable cells were affected by vitrification/warming, they were then similar or better after additional culture than in the fresh tissue. Proportions of cells labeled with differentiation markers also increased during in vitro culture. We demonstrated for the first time that black-footed ferret testicular tissues can be vitrified and revived, which will potentially contribute to new strategies to enhance assisted reproduction as well as conservation efforts in that rare and endangered species.

**Abstract:**

Systematic cryo-banking of semen and testicular tissues is critical to preserve the genetic value of recently deceased or neutered black-footed ferrets (BFFs). Specifically, recovering or producing mature sperm cells from vitrified-warmed issues offers additional options in assisted reproduction. This could, in turn, enhance the genetic management of this rare and endangered species over multiple generations. The objective of the study was to evaluate structural properties, DNA fragmentation, cell viability, and germ cell composition in vitrified testicular tissues from BFFs directly after warming or after warming plus a short in vitro culture period. Tissue biopsies from five adult BFFs were either kept fresh or vitrified with a standard protocol (using dimethylsulphoxide (DMSO) and glycerol) and warmed at 50 °C for 5 s. Some of the warmed samples were then cultured in vitro for 24 h. Fresh, warmed, and warmed/cultured tissues were analyzed using different indicators: histology of seminiferous tubules, intact Sertoli cells (vimentin labeling), DNA integrity, cell viability, germ cell composition (Oct4 and Boule labeling). Percentages of intact seminiferous tubules decreased after vitrification/warming and returned to the level of fresh samples after culture. While percentages of cells labeled with vimentin, with intact DNA integrity, or proportions of viable cells were affected by vitrification/warming, they all reached similar or better levels than the fresh tissue after culture. Proportions of cells labeled with Boule antibodies also improved during in vitro culture post-warming. We demonstrated for the first time that BFF testes subjected to vitrification, rapid warming, and short in vitro culture were viable and maintained the ability to resume germ cell progression. Cryopreserved testicular tissues could potentially contribute to new strategies to enhance BFF assisted reproduction as well as conservation efforts.

## 1. Introduction

The rescue and survival of the black-footed ferret (*Mustela nigripes*) (BFF) have heavily relied on extensive studies of the reproductive biology and the development of assisted reproductive technologies for more than 35 years [1]. This includes the establishment of optimized protocols for artificial insemination, semen cryopreservation, and systematic cryo-banking. BFFs are seasonal breeders, reaching puberty at the age of 1 year, followed by a relatively short reproductive life (3 to 4 years) [2]. Therefore, it is critical to collect or rescue and store genetic materials from all individuals for later use. As seen in other rare and endangered species, long-term preservation of ovarian and testicular tissues from individuals sterilized for medical reasons or dying unexpectedly offers additional options to study and propagate genetically valuable animals over multiple generations [3]. Cryopreservation of testicular tissues is now conducted in humans, laboratory models, domestic species, and some wildlife [3,4,5]. However, there is no report in the BFF yet.

Among the multiple preservation protocols that are available for testicular tissues, vitrification or ultra-rapid freezing is the most convenient approach that has already provided encouraging results for carnivore tissues regarding the maintenance of histomorphometry [3,6]. While cryoprotectants, exposure conditions, tissue biopsy size, or freezing rates play critical roles in tissue survival [3], warming is an essential step that is often overlooked. Proper warming can prevent devitrification/ice recrystallization and ensure optimal reanimation in preserved tissues. Recently, beneficial effects of short time exposure to high warming temperatures have been reported in testicular tissues from prepubertal cats [7]. An additional short culture period of warmed tissues also promotes cell survival and mitigates DNA injuries [7,8].

Interestingly, spermatozoa from frozen-thawed testicular tissues are viable in the cat model (based on the birth of healthy offspring after transfer of embryos produced by sperm injection) [9]. However, testicular tissues contain a large number of germ cells at early stages (in seminiferous tubules) that have the potential to develop into mature sperm cells after in vitro culture or xenografting as seen in other species, including the domestic ferret [3,10]. Spermatogenesis requires adequate germ cell support that may be compromised during freezing/warming as seen in humans [5] or in the cat model [6]. Sertoli cells are critical to fully support germ cell development and differentiation throughout spermatogenesis [11]. Thus, the structure and functions of seminiferous tubules, including connections between germ cells (histomorphometry) and the integrity of Sertoli cells (assessed by the presence of vimentin), have to be properly preserved [6,7]. Recent studies in the cat model confirmed that markers such as Oct4 and Boule are present in pre-meiotic (spermatogonia) and meiotic (spermatocytes) germ cells, respectively [7]. Those are very good indicators of germ cell composition in the tissue. However, the use of these critical markers to evaluate germ cell populations and progression has never been reported in BFFs.

Therefore, the objective of the study was to evaluate structural properties, cell survival, DNA fragmentation, and germ cell composition in vitrified testicular tissues from the endangered BFF directly after warming or after warming plus a short in vitro culture period.

## 2. Materials and Methods

### 2.1. Collection and Dissection of Testes

Tissues were sent to our laboratory for cryo-banking (collaboration with the US Fish and Wildlife Service’s National Black-Footed Ferret Conservation Center in Colorado). Testis and epididymis from BFFs (*n* = 5; 3–5 year old) were collected from routine orchiectomy of gonadal rescue (shortly after the unexpected death of the animal) from January to September 2018. The use of a subset of tissues for the present study was approved by the National Black-Footed Ferret Conservation Center and did not require approval from our Institutional Animal Care and Use Committee.

Testes were shipped overnight in phosphate buffered saline (PBS) at 4 °C to the laboratory. They were measured for length (L; cranial–caudal) and width (W; medial–lateral) and depth (D; dorso–ventral) using a caliper. Testes were extensively rinsed in PBS and dissected from surrounding tissues. Then, they were cut in pieces of approximately 1–3 mm^3^ using scalpel blades and forceps in Hepes–Ham’s F10 medium (Irvine Scientific, Santa Ana, CA, USA) supplemented with 1mM pyruvate, 2 mM L-glutamine, 100 IU/mL penicillin, 100 µg/mL streptomycin, 2.5% fetal bovine serum (FBS; Sigma-Aldrich, St Louis, MO, USA). A piece of epididymis was sliced with a scalpel blade in the same handling medium to detect the presence of spermatozoa.

### 2.2. Vitrification of Testicular Tissue

Tissue biopsies were immediately evaluated (fresh control; see Experimental Design) or exposed to a mix of cryoprotectants (dimethylsulphoxide (DMSO) and glycerol (Sigma- Aldrich) following our standard protocol [7]. Briefly, tissue biopsies were threaded onto a 30-G needle (BD Precision Glide needle, Fischer Scientific, Waltham, MA, USA) and immersed in an equilibrium (1.4 M of each cryoprotectant plus 0.25 M sucrose in Ham’s F10) for 10 min at room temperature (~22–24 °C). Tissues then were rapidly transferred to a vitrification solution (2.8 M of each cryoprotectant, 0.50 M sucrose, Ham’s F10, and 10% of FBS) for 5 min at room temperature. Threaded tissues then were plunged directly into liquid nitrogen and placed in sealed cryotubes before storage in a cryo-tank for at least one week.

### 2.3. Warming of Vitrified Tissues

Frozen tissue biopsies threaded onto needles were first transferred for 5 s into PBS solution pre-warmed to 50 °C. Progressive removal of the cryoprotectants was obtained by exposing tissues at room temperature to solutions containing decreasing concentrations of sucrose (0.50 M; 0.25 M; 0.00 M) in Hepes–Ham’s F10 and 20% of FBS for 5 min each.

### 2.4. Tissue Culture

Tissues were deposited on small cubes (1 cm^3^) of 1.5% agarose gel at the bottom of 4-well culture dishes filled with 400 µL of culture medium comprised of Hepes–Ham’s F10 (supplemented with 2 mM L-glutamine, 1 mM pyruvate, 100 IU/mL penicillin, 100 μg/mL streptomycin, and 5% FBS). Two tissue biopsies were placed on each cube and were cultured for 24 h at 38.5 °C in a humidified atmosphere with 5% CO^2^.

### 2.5. Histomorphology

Tissues were fixed overnight in Bouin’s solution before embedding in paraffin and serial sections (5-µm thick). After mounting sections on frosted glass slides, staining with hematoxylin-eosin was performed according to a standard protocol [7]. Slides then were observed using an upright microscope fitted with digital photomicrography (SPOT advanced software 5.0; Diagnostic Instruments, Sterling Heights, MI, USA). Seminiferous tubule and cell integrity were evaluated based on the attachment of cells to the basal membrane, absence of breaks in the stroma, no swelling of the lamina propria, and tight junctions between cells. Structures received a score of 1 in that case [7]. If any change was observed in the previous criteria, tubules received a score of 0. For each individual and for each treatment group, a total of 50 seminiferous tubules (randomly selected on the slides) were assessed. Percentages of normal seminiferous tubules were calculated relative to the total number of observed tubules.

### 2.6. Vimentin, Oct4, and Boule Immunostaining

Following a standard protocol [7], cells were isolated by slicing tissue pieces with a scalpel blade in modified Ham’s F-10 Basal Medium–HEPES (Irvine Scientific). Resulting cell suspensions were centrifuged (300× *g*) for 8 min and resuspended in fresh Ham’s F10 medium. After smearing 20 µL of suspension on a glass slide, cells were fixed in 4% paraformaldehyde for 1 h at room temperature followed by permeabilization with 0.1% Triton X-100 in PBS (PBS-T) for 3 min. After saturation of non-specific sites in PBS with 5% Bovine Serum Albumin (BSA, Sigma-Aldrich) for 1 h at room temperature, cells were incubated overnight at 4 °C in a humidified chamber with a primary antibody: anti-vimentin (1:500, Abcam #ab8069), pre-meiotic marker anti-OCT4 (1:200, Abcam #ab137427), or meiotic marker anti-Boule (1:100, Abcam #ab28745, Cambridge, MA, USA). After two washing in PBS and PBS-T for 5 min each, cells were incubated for 1 h at 37 °C with secondary antibodies: anti-mouse IgG (1:500, Invitrogen #62-6520, Carlsbad, CA, USA), donkey anti-goat IgG-FITC (1:500, Santa Cruz Biotechnology #2024, Dallas, TX, USA), or goat anti-rabbit IgG-TR (1:100; Santa Cruz Biotechnology #2780). Nuclear chromatin then was counterstained with Hoechst 33342 (1:100, Sigma-Aldrich) for 10 min at room temperature before mounting slides with Vectashield (Vector laboratories, Malvern, PA, USA). Slides were observed and images were captured using a microscope fitted with epifluorescence (Olympus BX41, Olympus Corporation, Cemter Valley, PA, USA) and with a SPOT camera and advanced software 5.0 (Diagnostic Instruments). For each individual and for each treatment group, 200 cells were observed for each staining. Percentages of stained cells were calculated relative to the total number of observed cells.

### 2.7. Evaluations of DNA Fragmentation and Viability

DNA integrity and cell viability were simultaneously assessed using the In-Situ Cell Death Detection kit (Roche, Burlington, NC, USA), as previously reported [7]. Briefly, cell suspensions were obtained as mentioned above. After smearing 20 µL of suspension on a glass slide, cells were fixed in 4% paraformaldehyde for 1 h at room temperature and then permeabilized on ice for 2 min in PBS with 0.1% Triton X-100 and 0.1% sodium citrate (PBS-T). After two washing in PBS, cells were incubated with Terminal deoxynucleotidyl transferase dUTP nick end labeling (TUNEL) reaction mixture (prepared according to the manufacturer) for 1 h at 37 °C in humid environment. A negative control slide (omission of terminal deoxynucleotidyl transferase, TdT, in the TUNEL reaction mixture) was included in each staining. A positive control slide was added in each staining as well by pre-incubating the cells in DNase I recombinant (Sigma-Aldrich) for 10 min before adding the TUNEL reaction mixture. Final counterstaining with Hoechst 33342 (1:100, Sigma-Aldrich; for nuclear chromatin) and propidium iodide (1:100, Invitrogen; for dead cell nuclei) lasted 10 min at room temperature before mounting slides in Vectashield (Vector laboratories). Slides were observed and images were captured using a microscope fitted with epifluorescence (Olympus BX41, Olympus Corporation) and with a SPOT camera and advanced software 5.0 (Diagnostic Instruments). Cells with DNA fragmentation had a bright green nucleus and dead cells had a bright red nucleus. For each individual and for each treatment group, we observed 200 cells. Proportions of cells with fragmented DNA were calculated relative to the total number of live cells. Percentages of viable cells were calculated based on the number of viable cells (normal DNA integrity or fragmented DNA) relative to the total number of cells.

### 2.8. Experimental Design and Statistical Analysis

On a given day, tissue biopsies from one individual were randomly allocated to different groups: control (fresh tissue), vitrification, or vitrification and culture. For histomorphological evaluation, 1-2 biopsies were fixed. For vimentin, DNA fragmentation, viability, Boule, or Oct4, 1-2 testicular pieces were dissected. Experiments were repeated five times (*n* = 5 individuals) on different days. Data were expressed as mean and standard error. All data analyses were conducted with statistical software GraphPad Prism^®^ version 5.01 (GraphPad Software Inc., San Diego, CA, USA). Data distribution was systematically tested with the Shapiro–Wilk test. When data distributions were normal, average proportions were compared between groups using analysis of variance (ANOVA) followed by a Tukey test. When data distributions were not normal, values were analyzed using a Wilcoxon or Mann Whitney test (non-parametrical test). Differences were considered statistically significant when *p* < 0.05.

## 3. Results

### 3.1. Testicular Morphometry

**Figure 1:**
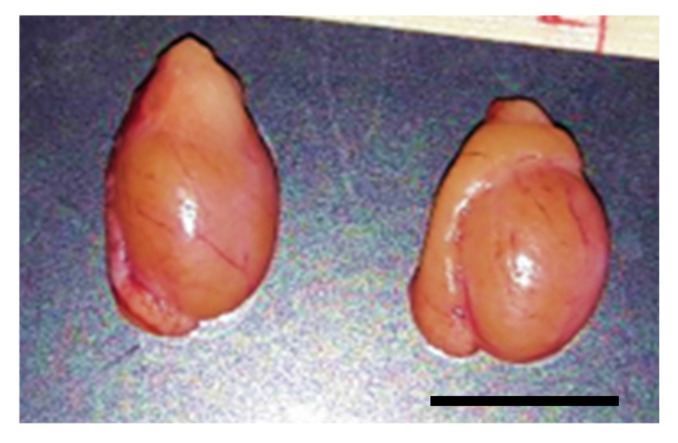
Testis pair (including epididymis) from an adult black-footed ferret. Bar = 1 cm.

Testes from different males had comparable sizes and volumes (Figure 1, Table 1). Motile spermatozoa were found in the epididymis of each male. Mature sperm cells also were occasionally found in the testicular tissues during dissection.

### 3.2. Influence of Vitrification and Culture on Tissue Structure and Viability

**Figure 2:**
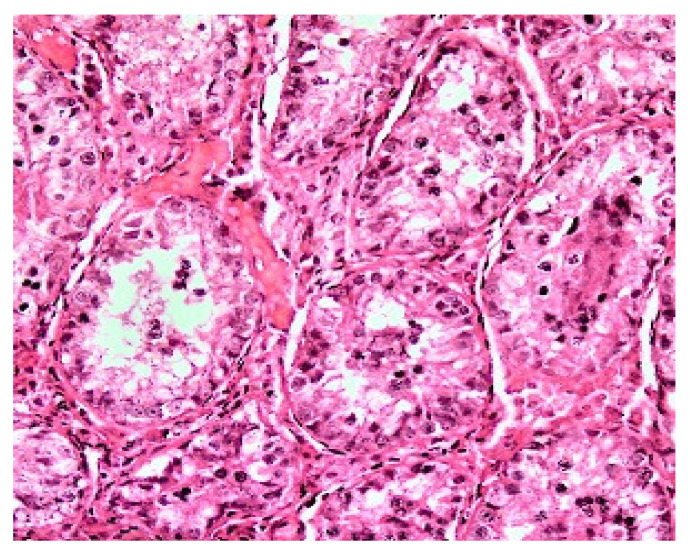
Histology of fresh testicular tissue (sections of seminiferous tubules) from an adult black-footed ferret. Bar = 20 µm.

**Figure 3:**
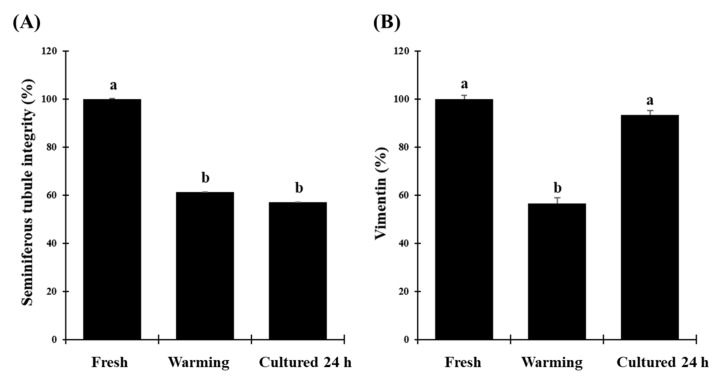
Morphological evaluation of fresh, vitrified/warmed (warming), or vitrified/warmed/cultured (cultured 24 h) testicular tissue from black-footed ferrets. (**A**) Proportion of intact seminiferous tubules, (**B**) proportion of Sertoli cells stained with vimentin. Data are expressed as mean ± SE (*n* = 5 animals per treatment). Different letters above bars indicate significant statistical differences between treatments (*p* < 0.05).

**Figure 4:**
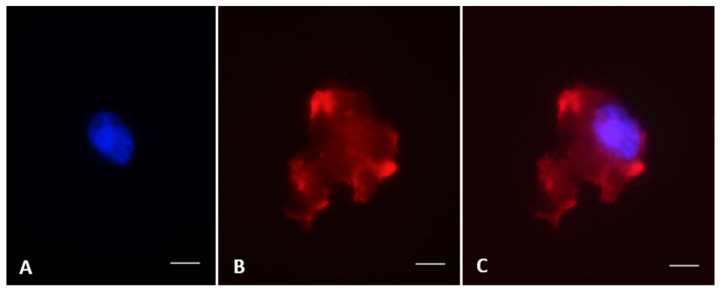
Immunostaining of vimentin in testicular cells from black-footed ferret. (**A**) Nucleus stained with Hoechst, (**B**) presence of vimentin in the cytoplasm, (**C**) merged image. Bar = 5 µm.

**Figure 5:**
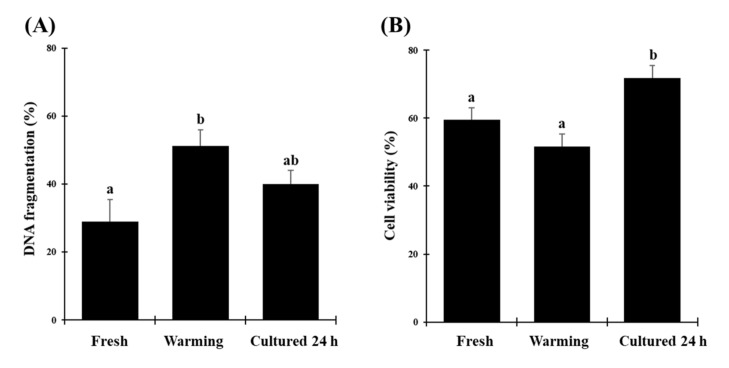
Proportions of cells with DNA fragmentation (**A**) and viable cells (**B**) in fresh, vitrified/warmed (warming), or vitrified/warmed/cultured (cultured 24 h) testicular tissues from black-footed ferrets. Data are expressed as mean ± SE (*n* = 5 animals per treatment). Different letters above bars indicate significant statistical differences between treatments (*p* < 0.05).

**Figure 6:**
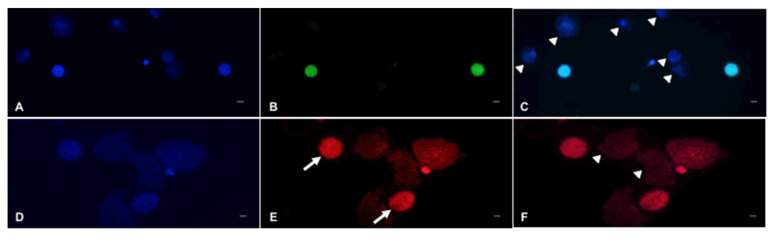
Representative cells with fragmented DNA or non-viable cells in testicular tissue from black-footed ferrets. (**A**,**D**) Nucleus stained with Hoechst, (**B**) live cells with fragmented DNA showing bright green nucleus, (**C**) merged image (white arrow heads indicate live cells with intact DNA), (**E**) dead cells showing bright red nucleus (arrows), (**F**) germ cells merged image (white arrow heads indicate live cells). Bar = 5 µm.

Proportions of intact seminiferous tubules were close to 100.0% in fresh tissues (Figure 2) but were lower (*p* < 0.05) after warming (61.2 ± 0.1%) and after in vitro culture (57.1 ± 0.1%; Figure 3A). However, there were no differences between warmed and cultured tissues (*p* > 0.05; Figure 3A). Percentages of cells labeled with vimentin after warming (56.5 ± 2.4%) were lower (*p* < 0.05) than in the fresh group (100.0 ± 1.5%). However, percentages of labeled cells after in vitro culture (93.2 ± 2.1%) did not differ from the fresh group (*p* > 0.05) and were significantly higher than the warmed tissues (Figure 3B and Figure 4). Proportions of cells with DNA fragmentation were higher (*p* < 0.05) in warmed (51.3 ± 4.7%) compared to the fresh (28.9 ± 6.5%) and cultured (40.0 ± 4.0%) tissues (Figure 5A and Figure 6). However, proportions of cells with DNA fragmentation in the fresh and cultured groups were similar (*p* > 0.05; Figure 5A and Figure 6). Percentages of viable cells increased (*p* < 0.05) after in vitro culture (71.8 ± 3.7%) compared to the fresh (59.5 ± 3.5%) and warmed (51.7 ± 3.6%) tissues (Figure 5B and Figure 6).

### 3.3. Influence of Vitrification and Culture on Germ Cell Composition

**Figure 7:**
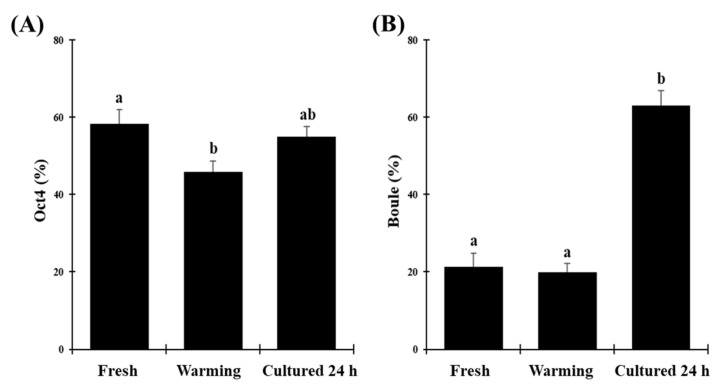
Evaluation of germ cell composition in fresh, vitrified/warmed (warming), or vitrified/warmed/cultured (cultured 24 h) testicular tissue from black-footed ferrets. (**A**) Proportion of cell stained with Oct4 (pre-meiotic stage), (**B**) Boule (meiotic stage). Data are expressed as mean ± SE (*n* = 5 animals per treatment). Different letters above bars indicate significant statistical differences between treatments (*p* < 0.05).

**Figure 8:**
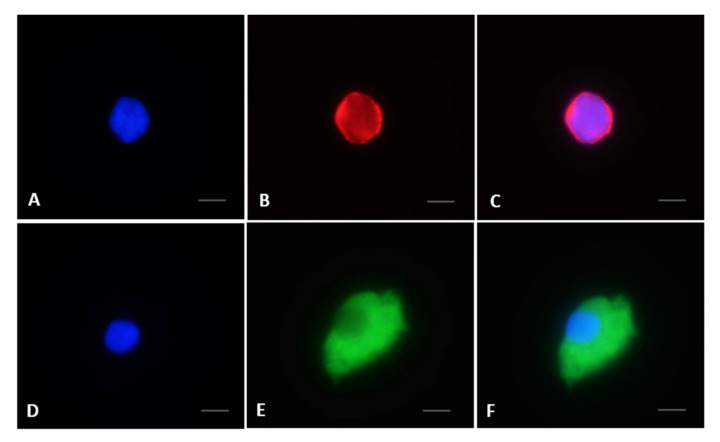
Immunostaining of Oct4 and Boule in testicular cells from black-footed ferrets. (**A**) Nucleus stained with Hoechst, (**B**) presence of Oct4 in the nucleus, (**C**) merged image, (**D**) nucleus stained with Hoechst, (**E**) presence of Boule in the cytoplasm, (**F**) merged image. Bar = 5 µm.

Proportions of germ cells labeled with OCT4 in fresh tissues (58.3 ± 3.7%) were higher compared to warmed groups (45.8 ± 2.9%; *p* < 0.05; Figure 7A and Figure 8). Cultured tissues led to percentages similar to fresh and warmed groups (55.0 ± 2.6%; *p* > 0.05; Figure 7A). Proportions of cells labeled with Boule were similar (*p* > 0.05) in the fresh (21.4 ± 3.5%) and warmed (19.9 ± 2.3%) groups (Figure 7B and Figure 8). However, the percentage of Boule-labeled cells increased significantly in cultured groups (63.0 ± 3.9%) compared to the other groups (*p* < 0.05; Figure 7B).

## 4. Discussion

This is the first report about testicular tissue cryopreservation in the BFF. Tissue pieces subjected to vitrification/warning followed by a short period of in vitro culture kept an overall normal structure and maintained the ability to resume germ cell progression.

Testicular morphometry and the presence of sperm cells in the tissue and the epididymis were comparable to previous reports in the same species [2]. Histology in fresh tissues also was similar to earlier studies conducted during the BFF breeding season [2]. Vimentin—a major component of the Sertoli cell cytoskeleton—was detected like in rodents, ungulates, or other carnivores [7,12,13]. The high prevalence of vimentin confirmed that critical cells supporting spermatogenesis were resilient and recovered after vitrification/warming plus culture as observed in the domestic cat [7].

Incidence of DNA fragmentation in testicular cells increased right after warming but returned to the same level after culture as in the fresh tissue. This likely involved DNA repair mechanisms during the additional culture period as previously observed in cat testes [7] but also in ovarian tissues exposed to comparable conditions [14]. Previous studies reported that germ cells at the early stages of spermatogenesis are more sensitive to DNA damage than sperm cells. Non-physiological conditions created by the preservation methods break hydrogen bonds between the bases of the DNA strands [15]. Our next step is to decipher and quantify DNA repair mechanisms in tissues through staining of proliferating cell nuclear antigen (PCNA) or RAD51 protein [16] and detection of heat shock proteins [17]. Similarly, testicular cell viability improved after culture like in other species, which is considered a good sign of functional reanimations [3]. Interestingly, viability after vitrification and culture was even better than in fresh tissues. We hypothesize that more cells had time to revive over 24 h of culture and eventually were present in higher proportions than in the refrigerated/fresh tissue shipped overnight to our laboratory. This is a phenomenon that has been already reported in gonadal tissues in many species (from ungulates to carnivores) following vitrification and warming [3,18].

The increase in Boule staining after culture was previously observed in domestic cat tissues [7]. This also was consistent with germ cell composition (progression from spermatogonia to spermatocytes) reported in the domestic ferret [19]. The higher incidence of Boule-labeled cells after culture could also be due to easier isolation of spermatocytes compared to the refrigerated/fresh tissues.

Present results were obtained in adult tissues, but it is expected that structural or functional properties in testes from prepubertal or younger BFFs would be comparable or even better, as seen in the preservation of cat testicular tissues [15]. Collective results helped to better characterize the resilience of BFF testicular tissues to freezing temperatures and the essential role of rapid warming followed by a 24-h reanimation period. These findings will have major implications for the development of cryo-banking in that species and future technologies involving the in vitro culture of testicular tissues (to produce mature sperm cells for in vitro fertilization, including sperm injection). It also is crucial to properly preserve testicular tissues because semen collection is not always possible (prepubertal individuals dying unexpectedly, castration for medical reasons during the non-breeding season, or obstructive azoospermia) [3].

## 5. Conclusions

Rapid warming and short-term culture enhanced survival and reanimation of vitrified testicular tissues. More experiments are warranted, but the lessons learned from this trial will pave the way for integrating biobanking and in vitro culture of testicular tissues in BFF conservation programs.

## Figures and Tables

**Table 1:** Testicular sizes of five adult black-footed ferrets during the 2018 breeding season.

Male Number	Collection Date	Size of Left Testis (L × W × D in mm)	Size of Right Testis (L × W × D in mm)
Male 1	February 2018	10 × 5 × 4	10 × 8 × 4
Male 2	April 2018	12 × 8 × 6	10 × 7 × 6
Male 3	April 2018	13 × 10 × 8	13 × 9 × 8
Male 4	May 2018	11 × 9 × 7	Damaged
Male 5	May 2018	13 × 10 × 10	13 × 10 × 8

## References

[1] Howard J.G., Lynch C., Santymire R.M., Marinari P.E., Wildt D.E. (2016). Recovery of gene diversity using long-term cryopreserved spermatozoa and artificial insemination in the endangered black-footed ferret. Anim. Conserv..

[2] Wolf K.N., Wildt D.E., Vargas A., Marinari P.E., Kreeger J.S., Ottinger M.A., Howard J.G. (2000). Age-dependent changes in sperm production, semen quality, and testicular volume in the black-footed ferret (Mustela nigripes). Biol. Reprod..

[3] da Silva A.M., Pereira A.F., Comizzoli P., Silva A.R. (2020). Cryopreservation and Culture of Testicular Tissues: An Essential Tool for Biodiversity Preservation. Biopreserv. Biobank..

[4] Comizzoli P. (2015). Biobanking efforts and new advances in male fertility preservation for rare and endangered species. Asian J. Androl..

[5] Schiewe M.C., Rothman C., Spitz A., Werthman P.E., Zeitlin S.I., Anderson R.E. (2016). Validation-verification of a highly effective, practical human testicular tissue in vitro culture-cryopreservation procedure aimed to optimize pre-freeze and post-thaw motility. J. Assist. Reprod. Genet..

[6] Lima D.B.C., da Silva T.F.P., Aquino-Cortez A., Leiva-Revilla J., da Silva L.D.M. (2018). Vitrification of testicular tissue from prepubertal cats in cryotubes using different cryoprotectant associations. Theriogenology.

[7] Lima D.B.C., Da Silva L.D.M., Comizzoli P. (2018). Influence of warming and reanimation conditions on seminiferous tubule morphology, mitochondrial activity, and cell composition of vitrified testicular tissues in the domestic cat model. PLoS ONE.

[8] Comizzoli P., Wildt D.E.D.E. (2013). Mammalian fertility preservation through cryobiology: Value of classical comparative studies and the need for new preservation options. Reprod. Fertil. Dev..

[9] Tharasanit T., Buarpung S., Manee-In S., Thongkittidilok C., Tiptanavattana N., Comizzoli P., Techakumphu M. (2012). Birth of kittens after the transfer of frozen-thawed embryos produced by intracytoplasmic sperm injection with spermatozoa collected from cryopreserved testicular tissue. Reprod. Domest. Anim..

[10] Gourdon J.C., Travis A.J. (2011). Spermatogenesis in ferret testis xenografts: A new model. Comp. Med..

[11] Nishimura H., L’Hernault S.W. (2017). Spermatogenesis. Curr. Biol..

[12] da Silva A.M., Bezerra L.G.P., Praxedes E.C.G., Moreira S.S.J., de Souza C.M.P., de Oliveira M.F., Pereira A.F., Comizzoli P., Silva A.R. (2019). Combination of intracellular cryoprotectants preserves the structure and the cells proliferative capacity potential of adult collared peccary testicular tissue subjected to solid surface vitrification. Cryobiology.

[13] Zomer H.D., Reddi P.P. (2020). Characterization of rodent Sertoli cell primary cultures. Mol. Reprod. Dev..

[14] Mouttham L., Comizzoli P. (2016). The preservation of vital functions in cat ovarian tissues during vitrification depends more on the temperature of the cryoprotectant exposure than on the sucrose supplementation. Cryobiology.

[15] Silva H.V.R., da Silva A.M., Lee P.-C., Brito B.F., Silva A.R., da Silva L.D.M., Comizzoli P. (2020). Influence of Microwave-Assisted Drying on Structural Integrity and Viability of Testicular Tissues from Adult and Prepubertal Domestic Cats. Biopreserv. Biobank..

[16] Mouttham L., Fortune J.E., Comizzoli P. (2015). Damage to fetal bovine ovarian tissue caused by cryoprotectant exposure and vitrification is mitigated during tissue culture. J. Assist. Reprod. Genet..

[17] Sottile M.L., Nadin S.B. (2018). Heat shock proteins and DNA repair mechanisms: An updated overview. Cell Stress Chaperones.

[18] Campos L.B., Praxedes É.C.G., Saraiva M.V.A., Comizzoli P., Silva A.R. (2019). Advances and challenges of using ovarian preantral follicles to develop biobanks of wild mammals. Biopreserv. Biobank..

[19] Nakai M., Van Cleeff J.K., Bahr J.M. (2004). Stages and duration of spermatogenesis in the domestic ferret (Mustela putorius furo). Tissue Cell.

